# Astrocyte biomarker signatures of amyloid-β and tau pathologies in Alzheimer’s disease

**DOI:** 10.1038/s41380-022-01716-2

**Published:** 2022-08-10

**Authors:** João Pedro Ferrari-Souza, Pâmela C. L. Ferreira, Bruna Bellaver, Cécile Tissot, Yi-Ting Wang, Douglas T. Leffa, Wagner S. Brum, Andréa L. Benedet, Nicholas J. Ashton, Marco Antônio De Bastiani, Andréia Rocha, Joseph Therriault, Firoza Z. Lussier, Mira Chamoun, Stijn Servaes, Gleb Bezgin, Min Su Kang, Jenna Stevenson, Nesrine Rahmouni, Vanessa Pallen, Nina Margherita Poltronetti, William E. Klunk, Dana L. Tudorascu, Ann D. Cohen, Victor L. Villemagne, Serge Gauthier, Kaj Blennow, Henrik Zetterberg, Diogo O. Souza, Thomas K. Karikari, Eduardo R. Zimmer, Pedro Rosa-Neto, Tharick A. Pascoal

**Affiliations:** 1 Department of Psychiatry, University of Pittsburgh, Pittsburgh, PA USA; 2 Graduate Program in Biological Sciences: Biochemistry, Universidade Federal do Rio Grande do Sul, Porto Alegre, RS Brazil; 3 Translational Neuroimaging Laboratory, McGill University Research Centre for Studies in Aging, Alzheimer’s Disease Research Unit, Douglas Research Institute, Le Centre intégré universitaire de santé et de services sociaux (CIUSSS) de l’Ouest-de-l’Île-de-Montréal; Department of Neurology and Neurosurgery, Psychiatry and Pharmacology and Therapeutics, McGill University, Montreal, QC Canada; 4 ADHD Outpatient Program & Development Psychiatry Program, Hospital de Clínicas de Porto Alegre, Porto Alegre, RS Brazil; 5 Department of Psychiatry and Neurochemistry, The Sahlgrenska Academy at the University of Gothenburg, Mölndal, Sweden; 6 Clinical Neurochemistry Laboratory, Sahlgrenska University Hospital, Gothenburg, Sweden; 7 Wallenberg Centre for Molecular and Translational Medicine, University of Gothenburg, Gothenburg, Sweden; 8 Department of Old Age Psychiatry, Institute of Psychiatry, Psychology & Neuroscience, King’s College London, London, UK; 9 Department of Neurodegenerative Disease, UCL Queen Square Institute of Neurology, London, UK; 10 UK Dementia Research Institute at UCL, London, UK; 11 Hong Kong Center for Neurodegenerative Diseases, Hong Kong, China; 12 Department of Pharmacology, Universidade Federal do Rio Grande do Sul, Porto Alegre, RS Brazil; 13 Graduate Program in Biological Sciences: Pharmacology and Therapeuctis, Universidade Federal do Rio Grande do Sul, Porto Alegre, RS Brazil

**Keywords:** Biomarkers, Diseases, Neuroscience

## Abstract

Astrocytes can adopt multiple molecular phenotypes in the brain of Alzheimer’s disease (AD) patients. Here, we studied the associations of cerebrospinal fluid (CSF) glial fibrillary acidic protein (GFAP) and chitinase-3-like protein 1 (YKL-40) levels with brain amyloid-β (Aβ) and tau pathologies. We assessed 121 individuals across the aging and AD clinical spectrum with positron emission tomography (PET) brain imaging for Aβ ([^18^F]AZD4694) and tau ([^18^F]MK-6240), as well as CSF GFAP and YKL-40 measures. We observed that higher CSF GFAP levels were associated with elevated Aβ-PET but not tau-PET load. By contrast, higher CSF YKL-40 levels were associated with elevated tau-PET but not Aβ-PET burden. Structural equation modeling revealed that CSF GFAP and YKL-40 mediate the effects of Aβ and tau, respectively, on hippocampal atrophy, which was further associated with cognitive impairment. Our results suggest the existence of distinct astrocyte biomarker signatures in response to brain Aβ and tau accumulation, which may contribute to our understanding of the complex link between reactive astrogliosis heterogeneity and AD progression.

## Introduction

Reactive astrocytes play an important role in Alzheimer’s disease (AD) pathophysiology [1–5]. *Post-mortem* studies suggest that both amyloid-β (Aβ) and tau pathologies are associated with astrocyte reactivity [1, 6]. Far from displaying a homogenous response, transcriptomics analyses demonstrated that reactive astrocytes can acquire multiple molecular phenotypes in the AD brain [7]. The context-specific aspects of astrocyte reactivity [8] raise the possibility that astrocytes respond differently to AD-related brain processes. In fact, experimental evidence indicates the presence of distinct molecular astrocyte signatures in response to Aβ and tau pathologies [9, 10]. However, knowledge about Aβ- and tau-specific contributions to reactive astrocyte biomarkers in patients with AD is still limited.

Reactive astrocytes overexpress specific proteins that can be released into the extracellular compartment, being measured in the cerebrospinal fluid (CSF) of living individuals [8, 11]. CSF levels of glial fibrillary acidic protein (GFAP) and chitinase-3-like protein 1 (YKL-40), biomarkers of astrocyte reactivity [8, 11], are consistently elevated in the dementia phase of AD [12], and in some other brain disorders such as multiple sclerosis [13, 14]. Although GFAP and YKL-40 fluid concentrations have already been shown to correlate with AD pathophysiology [15–20], no previous study has investigated the existence of Aβ- and tau-related astrocyte responses in the human brain. Identifying astrocyte biomarker signatures related to AD proteinopathies has the potential to provide insights into the role of astrocytes in disease progression, allow disease staging, and can lead to the development of drugs targeting distinct reactive astrocyte phenotypes.

In a cohort of individuals across the aging and AD clinical spectrum, we tested whether CSF GFAP and YKL-40 are distinctly associated with Aβ and tau pathologies. We also investigated whether these reactive astrocyte biomarkers mediate the effects of AD hallmark pathologies on neurodegeneration and cognitive impairment.

## Materials and methods

### Participants

Study participants are part of the Translational Biomarkers in Aging and Dementia (TRIAD) cohort, McGill University, Canada (https://triad.tnl-mcgill.com). Participants from the community or outpatients at the McGill University Research Centre for Studies in Aging were recruited through different sources, such as printed materials, word of mouth, and referrals. Exclusion criteria included inability to speak English or French, inadequate visual and auditory capacities for neuropsychologic assessment, active substance abuse, major surgery, recent head trauma, medical contraindication for positron emission tomography (PET) or magnetic resonance imaging (MRI), currently being enrolled in other studies, and neurological, psychiatric, or systemic comorbidities that were not adequately treated with a stable medication regimen. The Douglas Mental Health University Institute Research Ethics Board and the Montreal Neurological Instituted PET working committee approved this study. All participants provided written informed consent.

We assessed 75 cognitively unimpaired (CU) and 46 cognitively impaired [CI; 29 with mild cognitive impairment (MCI) and 17 with AD dementia] participants with 50 years of age or older. In addition to CSF GFAP and YKL-40, individuals had available Aβ-PET, tau-PET, and MRI at the same visit, as well as apolipoprotein E (*APOE*) genotyping. The mean time difference between CSF collection and imaging was 2.69 months (range: 0–11.7 months). There were 39 participants (25 CU, 8 with MCI, and 6 with AD dementia) with a time lag higher than 3 months between imaging and CSF collection. Supplementary Table 1 shows the time differences between imaging modalities. Two outliers were detected [CSF GFAP levels that were three standard deviations (SD) above the mean of the whole population] and excluded from subsequent analyses, as previously done [21, 22]. For a detailed description of the selection of study participants, see Supplementary Fig. 1. All individuals had detailed neuropsychological testing, including Mini-Mental State Examination (MMSE) and Clinical Dementia Rating (CDR). CU subjects had a CDR of 0 and no objective cognitive impairment. MCI patients had CDR of 0.5, subjective and objective cognitive impairments, and preserved activities of daily living [23]. Mild-to-moderate AD dementia patients had CDR of between 0.5 and 2 and met the National Institute on Aging and the Alzheimer’s Association (NIA-AA) criteria for probable AD [24]. In accordance with the updated 2018 NIA-AA Research Framework [25], AD dementia participants were required to be Aβ positive, similar to previous publications [26, 27]. Biomarkers were analyzed blinded to clinical diagnosis.

### Fluid biomarkers

All samples were analyzed at the Clinical Neurochemistry Laboratory at the University of Gothenburg, Sweden. CSF and plasma GFAP were quantified using a commercial single-plex assay (No. 102336) on the Single molecule array (Simoa) HD-X (Quanterix, Billerica, MA, USA) [16]. CSF YKL-40 was measured using a commercial ELISA assay (R&D Systems, Minneapolis, MN, USA) [16]. Moreover, a subset of 62 participants had CSF samples analyzed using a multiplex immunoassay for a panel of 92 proteins related to inflammatory diseases and associated biological processes (Olink; https://www.olink.com/products/inflammation/). We excluded 37 markers with a high percentage (>15%) of values below the lower detection limit, as previously described [28].

### Neuroimaging biomarkers

T_1_-weighted MRIs were acquired on a 3T Siemens Magnetom using a standard head coil, and the magnetization prepared rapid acquisition gradient echo (MPRAGE) sequence was used to obtain high-resolution structural images of the whole brain. Aβ-PET ([^18^F]AZD4694; 40–70 min post-injection) and tau-PET ([^18^F]MK-6240; 90–110 min post-injection) scans were acquired on a Siemens high-resolution research tomograph. Radiosynthesis of PET tracers have been described elsewhere [29, 30]. [^18^F]AZD4694 had a mean injected dose of 240.3 (SD = 20.9) MBq, and [^18^F]MK-6240 had a mean injected dose of 228.8 (SD = 34.7) MBq. Aβ-PET and tau-PET scans were reconstructed using the ordered subset expectation maximization algorithm on a 4D volume with three frames (3 × 600 s) and four frames (4 × 300 s), respectively [29]. Details regarding MRI and PET acquisition and processing are described in the Supplementary Methods.

We used the Desikan-Killiany-Tourville atlas to define the regions of interest (ROIs) [31]. For Aβ-PET, a global neocortical standardized uptake value ratio (SUVR) was estimated from the following brain regions: precuneus, prefrontal, orbitofrontal, parietal, temporal, and cingulate cortices [32]. Aβ (A) positivity was defined as neocortical Aβ-PET SUVR ≥ 1.55 following a published threshold for [^18^F]AZD4694 Aβ-PET [33]. Based on the standard [^18^F]AZD4694 dataset obtained from the Global Alzheimer’s Association Interactive Network (GAAIN; http://www.gaain.org), we calculated that the Aβ-PET SUVR threshold value of 1.55 corresponds to 24 Centiloid units [34, 35]. For tau-PET, a temporal meta-ROI SUVR was estimated from the following brain regions: entorhinal, hippocampus, fusiform, parahippocampal, inferior temporal, and middle temporal [32]. Tau (T) positivity was defined as temporal meta-ROI tau-PET SUVR ≥ 1.24, as described elsewhere [36]. Of note, there were no A − T+ subject in our analyses as we did not include Aβ negative individuals with a clinical diagnosis of AD dementia. Hippocampal volume was adjusted for total intracranial volume with a previously described residual approach [37, 38], which uses a linear regression between the raw hippocampal volume and the total intracranial volume in the CU Aβ negative group to calculate the TIV-adjusted hippocampal volume.

### Cognition

Cognition was assessed using the MMSE, as well as memory and executive composite scores. As previously described, the memory composite score was calculated as the average of four z-scores: Rey Auditory Verbal Learning Test (RAVLT) immediate recall, RAVLT delayed recall, Logical Memory immediate recall, and Logical Memory delayed recall [39]. The executive composite score was calculated as the average of three z-scores: Delis–Kaplan Executive Function System (D-KEFS) letter fluency, Trail Making Test B time, and Wechsler Adult Intelligence Scale - Third Edition (WAIS-III) digit span [39]. Z-scoring of raw test scores used in the memory and executive composites was conducted using the mean and standard deviation of CU elderly.

### Statistical analysis

Statistical analyses were conducted in the R free software (version 4.0.2, http://www.r-project.org/) for non-imaging analyses, and MATLAB software (version 9.2, http://www.mathworks.com) with VoxelStats package for imaging analyses [40]. Student’s *t* test (continuous variables) and contingency *χ*^2^ test (categorical variables) tested demographic differences. The association of biomarkers with disease severity was tested using regression analyses adjusting for age, sex, and *APOE* ε4 status. Global CDR score was categorized as 0 (no symptoms), 0.5 (very mild symptoms), and ≥1 (up to 2; mild-to-moderate symptoms). Spearman rank test was used to assess the correlations between reactive astrocyte biomarkers. Analysis of variance with Tukey’s multiple comparisons test was used to compare adjusted levels of GFAP and YKL-40 markers across groups defined based on Aβ-PET and tau-PET status. Adjusted values were the residuals of the regressions between biomarker level and covariates of interest (age, sex, and *APOE* ε4 status). The associations of Aβ-PET and tau-PET with reactive astrocyte biomarkers were tested using ROI-based linear regressions, as well as voxel-wise linear regressions. Models were adjusted for age, sex, cognitive status, and *APOE* ε4 status. In ROI-based multiple regression models, partial residuals generated with the R function termplot were used to graphically represent the association between two variables while adjusting for the other independent predictors [41, 42]. In voxel-wise analyses, multiple comparisons correction was performed using random field theory (RFT) [43], with a voxel threshold of *P* < 0.001. We evaluated whether CSF GFAP and YKL-40 mediate the effect of Aβ and tau pathologies on neurodegeneration and cognition using structural equation modeling, R package “lavaan” [44]. The fit of the structural equation models was classified as good if: comparative fit index (CFI) > 0.97 (acceptable: 0.95–0.97); root mean squared error of approximation (RMSEA) < 0.05 (acceptable 0.05–0.08); standardized root mean square residual (SRMR) < 0.05 (acceptable: 0.05–0.10) [45, 46]. Continuous variables were standardized in regression and structural equation models to facilitate the interpretation of our findings and allow the direct comparison between estimates. The association of CSF GFAP and YKL-40 with CSF inflammation-related proteins was assessed with age-adjusted partial correlations with Bonferroni correction for multiple comparisons. Furthermore, the Search Tool for the Retrieval of Interacting Genes/Proteins (STRING) database (version 11.5) [47] was used to construct a protein-protein interaction network including GFAP and YKL-40, as well as the inflammation-related proteins that were significantly correlated to these reactive astrocyte biomarkers. For graphical representation, we filtered connections with STRING confidence interaction scores > 0.4. The statistical significance level was set as *P* < 0.05, two-tailed.

## Results

Demographic information of the population can be found in Table 1. No statistically significant difference was observed between CU and CI groups regarding age, sex, and years of education. CI subjects had lower MMSE scores, higher neocortical Aβ-PET SUVR, higher temporal meta-ROI tau-PET SUVR, and lower hippocampal volume than CU subjects. Additionally, individuals in the CI group were more likely to be *APOE* ε4 carriers compared to individuals in the CU group. CSF and plasma GFAP levels were significantly higher in individuals with a CDR score of 0.5 (CSF GFAP: *P* = 0.043; plasma GFAP: *P* = 0.010) and with a CDR score ≥1 (CSF GFAP: *P* = 0.029; plasma GFAP: *P* < 0.001) compared to individuals with a CDR score of 0. CSF YKL-40 levels were significantly higher in subjects with a CDR score ≥1 (*P* = 0.008) but not with a CDR of 0.5 (*P* = 0.686) in comparison to subjects with a CDR score of 0. We also observed that both CSF and plasma GFAP were negatively associated with the memory composite score (CSF GFAP: *β* = −0.29, *P* = 0.022; plasma GFAP: *β* = −0.55, *P* < 0.001) but not with the executive composite score (CSF GFAP: *β* = −0.09, *P* = 0.335; plasma GFAP: *β* = −0.17, *P* = 0.074). On the other hand, CSF YKL-40 was negatively associated with both the memory (*β* = −0.40, *P* = 0.002) and executive (*β* = −0.22, *P* = 0.020) composite scores. Additionally, the interaction between Aβ and tau was not associated with disease severity (Supplementary Tables 2 and 3). Average and SD Aβ-PET and tau-PET SUVR maps are presented in Supplementary Fig. 2. Correlations between reactive astrocyte biomarkers are presented in Supplementary Fig. 3.

**Table 1:** Demographics and key characteristics of participants by cognitive status.

	CU	CI	*P* value
No.	75	46	–
Age, years	70.9 (5.8)	68.9 (7.6)	0.136
Male, No. (%)	28 (37.3)	25 (54.3)	0.101
Education, years	14.7 (3.4)	15.3 (3.1)	0.359
*APOE* ε4 carriers, No. (%)	22 (29.3)	26 (56.5)	0.006
MMSE score	29.1 (1.0)	25.2 (5.4)	<0.001
Neocortical Aβ-PET SUVR	1.51 (0.4)	2.20 (0.6)	<0.001
Temporal meta-ROI tau-PET SUVR	0.86 (0.1)	1.59 (0.8)	<0.001
Hippocampal volume, cm^3^	3.53 (0.4)^a^	3.16 (0.5)^a^	<0.001

Continuous variables are presented as mean (SD).

*Aβ* amyloid-β, *APOE* ε4 Apolipoprotein E ε4, *CI* cognitively impaired, *CU* cognitively unimpaired, *MMSE* Mini-Mental State Examination, *PET* positron emission tomography, *ROI* region of interest, *SD* standard deviation, *SUVR* standardized uptake value ratio.

^a^Values reported are adjusted for total intracranial volume.

### Higher GFAP levels are associated with Aβ positivity and YKL-40 levels with tau positivity

**Fig. 1:**
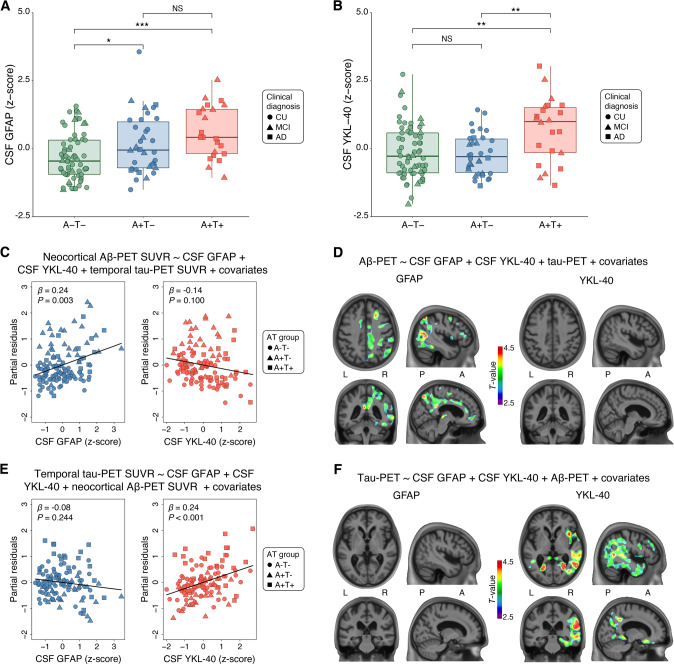
GFAP associates with Aβ and YKL-40 with tau accumulation. The panels show box-and-whisker plots of **A** CSF GFAP and **B** CSF YKL-40 levels adjusted for age-, sex-, and *APOE* ε4 status across AT groups. The horizontal line in each box represents the median; box ends represent the 25th and 75th percentiles. Shape of the dots depicts the clinical diagnosis (CU: 73.3% A-T-, and 26.7% A + T−; MCI: 34.5% A − T−, 34.5% A + T−, and 31.0% A + T+; AD: 23.5% A + T−, and 76.5% A + T). Groups were compared using analyses of variance with Tukey’s multiple comparison test (*P < 0.05, **P < 0.01, ***P < 0.001). **C** Partial residual plots of ROI-based linear regressions testing the associations of neocortical Aβ-PET SUVR with CSF GFAP and YKL-40 levels adjusting for temporal meta-ROI tau-PET SUVR. The shape of the dots depicts the AT group. **D** *T*-statistical parametric maps show the result of voxel-wise linear regression testing the regional association of Aβ-PET SUVR with CSF GFAP and YKL-40 levels adjusting for tau-PET SUVR. R and L indicate right and left, respectively; A and P denote anterior and posterior, respectively. **E** Partial residual plots of ROI-based linear regressions testing the associations of temporal meta-ROI tau-PET SUVR with CSF GFAP and YKL-40 levels adjusting for neocortical Aβ-PET SUVR. The shape of the dots depicts the AT group. **F** *T*-statistical parametric maps show the result of voxel-wise linear regression testing the regional association of tau-PET SUVR with CSF GFAP and YKL-40 levels adjusting for neocortical Aβ-PET SUVR. R and L indicate right and left, respectively; A and P denote anterior and posterior, respectively. Voxel-wise linear regressions were RFT-corrected for multiple comparisons at a voxel threshold of *P* < 0.001. Age, sex, cognitive status, and *APOE* ε4 status were used as covariates for adjustment in all ROI- and voxel-based linear regressions. NS not significant.

**Fig. 2:**
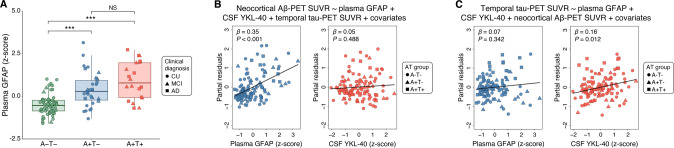
Sensitivity analyses testing the associations of Aβ-PET and tau-PET with reactive astrocyte biomarkers using plasma GFAP instead of CSF GFAP. **A** Box-and-whisker plot of plasma GFAP levels adjusted for age, sex, and *APOE* ε4 status across AT groups. The horizontal line in each box represents the median; box ends represent the 25th and 75th percentiles. Shape of the dots depicts the clinical diagnosis (CU: 73.6% A-T-, and 26.4% A + T−; MCI: 37.0% A − T−, 29.6% A + T−, and 33.3% A + T+; AD: 26.7% A + T−, and 73.3% A + T). Groups were compared using analyses of variance with Tukey’s multiple comparison test (*P < 0.05, **P < 0.01, ***P < 0.001). **B** Partial residual plots of linear regressions testing the associations of neocortical Aβ-PET SUVR with plasma GFAP and CSF YKL-40 levels adjusting for temporal meta-ROI tau-PET SUVR, age, sex, cognitive status, and *APOE* ε4 status. The shape of the dots depicts the AT group. **C** Partial residual plots of linear regressions testing the associations of temporal meta-ROI tau-PET SUVR with plasma GFAP and CSF YKL-40 levels adjusting for neocortical Aβ-PET SUVR, age, sex, cognitive status, and *APOE* ε4 status. The shape of the dots depicts the AT group. Of note, analyses involving plasma GFAP were conducted in a subset of 114 individuals; from the total study population of 121 subjects, five participants did not have available plasma GFAP measures, and two were excluded because they were considered outliers (plasma GFAP concentrations three SD above the mean of the population). NS not significant.

We assessed the levels of reactive astrocyte biomarkers across groups defined by Aβ-PET (A) and tau-PET (T) status. CSF GFAP levels were significantly higher in A + T- and A + T+ groups compared with the A-T- group (A + T− vs. A − T−: *P* = 0.048; A + T+ vs. A − T−: *P* < 0.001; Fig. 1A). Furthermore, no statistically significant difference was observed between A + T− and A + T+ groups (*P* = 0.196; Fig. 1A). In relation to CSF GFAP, similar findings were observed for plasma GFAP levels across groups (Fig. 2A). CSF YKL-40 levels were higher in the A + T+ group as compared with A − T− (*P* = 0.006) and A + T− (*P* = 0.004) groups (Fig. 1B). Moreover, no significant difference was observed when comparing A − T− and A + T− groups (*P* = 0.868; Fig. 1B). Biomarker levels of 3 A − T+ dementia individuals that were excluded from the analysis and had CSF YKL-40 available are presented in Supplementary Fig. 4.

### GFAP but not YKL-40 is associated with Aβ-PET burden

We investigated the associations of Aβ-PET burden with CSF GFAP and YKL-40 levels adjusting for tau-PET SUVR, age, sex, cognitive status, and *APOE* ε4 status. ROI-based linear regression model revealed that higher CSF GFAP but not CSF YKL-40 levels were associated with higher neocortical Aβ-PET SUVR (CSF GFAP: *β* = 0.24, *P* = 0.003; CSF YKL-40: *β* = −0.14, *P* = 0.100; Fig. 1C and Model A in Supplementary Table 4). In additional analyses excluding individuals with a time lag higher than 3 months between imaging and CSF collection, we observed similar results (Model A in Supplementary Table 5). Voxel-wise analysis showed that CSF GFAP levels were positively associated with Aβ-PET load in Aβ-related brain regions (e.g., precuneus, cingulate, orbitofrontal, and lateral temporal), mainly in the right hemisphere (Fig. 1D). No association was detected between CSF YKL-40 concentrations and Aβ-PET SUVR (Fig. 1D). Voxel-wise results before multiple comparisons correction are shown in Supplementary Fig. 5A. We also conducted sensitivity analysis using plasma GFAP instead of CSF GFAP. We observed that plasma GFAP but not CSF YKL-40 levels were positively associated with Aβ-PET burden (plasma GFAP: *β* = 0.35, *P* < 0.001; CSF YKL-40: *β* = 0.05, *P* = 0.488; Fig. 2B), reinforcing the aforementioned results.

### YKL-40 but not GFAP is associated with tau-PET burden

We further tested the associations of tau-PET uptake with CSF GFAP and YKL-40 levels adjusting for Aβ-PET SUVR, age, sex, cognitive status, and *APOE* ε4 status. ROI-based regressions demonstrated that higher CSF YKL-40 but not CSF GFAP levels were associated with higher temporal meta-ROI tau-PET SUVR (CSF GFAP: β = −0.08, *P* = 0.244; CSF YKL-40: *β* = 0.24, *P* < 0.001; Fig. 1E and Model B in Supplementary Table 4). Similar results were observed when excluding individuals with a time lag higher than 3 months between imaging and CSF collection (Model B in Supplementary Table 5). Voxel-wise linear regression analysis demonstrated that CSF YKL-40 levels were positively associated with tau-PET uptake in early and late Braak regions, mainly in the right hemisphere (Fig. 1F). No association was observed between CSF GFAP levels and tau-PET uptake (Fig. 1F). Voxel-wise linear regression results before correction for multiple comparisons are displayed in Supplementary Fig. 5B. In sensitivity analysis using plasma GFAP instead of CSF GFAP, we found that higher CSF YKL-40 but not plasma GFAP levels were associated with higher tau-PET burden (plasma GFAP: *β* = 0.07, *P* = 0.342; CSF YKL-40: *β* = 0.16, *P* = 0.012; Fig. 2C), which supports the aforementioned results. The overlap between the brain regions showing a significant association of CSF GFAP and YKL-40 with Aβ-PET and tau-PET, respectively, is shown in Supplementary Fig. 6.

### GFAP and YKL-40 mediate hippocampal atrophy and cognitive impairment

**Fig. 3:**
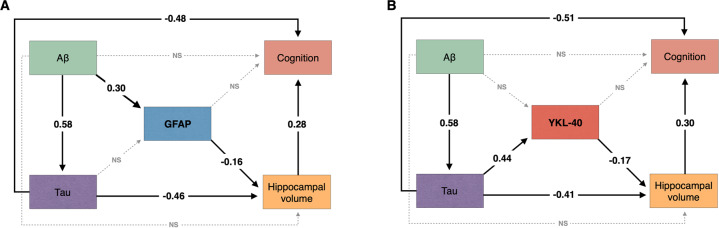
Reactive astrocyte biomarkers mediate the effect of AD pathophysiology on downstream neurodegeneration and cognitive impairment. The figure shows the standardized structural equation model estimates of the associations between **A** CSF GFAP and **B** CSF YKL-40 levels, hippocampal volume, and cognition. Solid black lines represent significant associations, whereas dashed gray lines represent non-significant effects. The fact that the presented estimates are standardized allows direct comparison between effects. Aβ pathology was measured with neocortical Aβ-PET SUVR and tau pathology using temporal meta-ROI tau-PET SUVR. Cognition was indexed using the MMSE score. All associations were further adjusted for age, *APOE* ε4 status, and years of education. NS not significant.

We tested whether CSF GFAP mediates the association of AD pathophysiology with hippocampal atrophy and cognitive impairment using structural equation modeling. The model demonstrated that CSF GFAP levels partially mediate the effect of higher Aβ-PET on lower hippocampal volume. We observed an indirect effect of Aβ pathology on cognition through higher CSF GFAP levels and lower hippocampal volume (Fig. 3A). The aforementioned structural equation model fitted the data well (CFI = 1.00; RMSEA = 0.00; SRMR = 0.00). See Supplementary Table 6 for complete model coefficients and associated statistics.

Furthermore, we tested whether CSF YKL-40 mediates the association of AD pathophysiology with hippocampal atrophy and cognitive impairment using structural equation modeling. The model revealed that higher tau-PET uptake was associated with lower hippocampal volume directly as well as indirectly through higher CSF YKL-40 levels. The model suggests that tau effects on cognitive impairment were partially mediated through higher CSF YKL-40 levels and lower hippocampal volume (Fig. 3B). The aforementioned structural equation model yielded a robust fit (CFI = 1.00; RMSEA = 0.00; SRMR = 0.00). See Supplementary Table 7 for model coefficients and associated statistics.

### Reactive astrocyte biomarkers are associated with brain inflammation

In a subgroup of 62 participants (36 CU and 26 CI), we assessed the associations of CSF GFAP and YKL-40 with several inflammation-related proteins. Entire list of CSF inflammatory proteins with the corresponding abbreviations is reported in Supplementary Table 8. Demographics for the subgroup of participants can be found in Supplementary Table 9. No significant differences were observed when comparing the demographic information between the subsample of individuals with available CSF inflammatory markers and the total population included in the present study (Supplementary Table 10). We observed that both CSF GFAP and YKL-40 were positively correlated with CSF inflammatory markers, including eukaryotic translation initiation factor 4E-binding protein 1 (4E-BP1), macrophage colony-stimulating factor 1 (CSF-1), fractalkine (CX3CL1), FMS-related tyrosine kinase 3 ligand (Flt3L), hepatocyte growth factor (HGF), interleukin-10 receptor subunit beta (IL-10RB), leukemia inhibitory factor receptor (LIF-R), matrix metalloproteinase-10 (MMP-10), stem cell factor (SCF), STAM-binding protein (STAMBP), transforming growth factor alpha (TGF-alpha), TNF-related apoptosis-inducing ligand (TRAIL), tumor necrosis factor ligand superfamily member 12 (TWEAK), and urokinase-type plasminogen activator (uPA) (Supplementary Fig. 7A, B). Additionally, CSF YKL-40 but not CSF GFAP was also positively correlated with CD40L receptor (CD40), T-cell surface glycoprotein CD5 (CD5), C-X-C motif chemokine 1 (CXCL1), interleukin-12 subunit beta (IL-12B), interleukin-8 (IL-8), SIR2-like protein 2 (SIRT2), and vascular endothelial growth factor A (VEGF-A; Supplementary Fig. 7A, B). No significant negative associations of CSF GFAP and YKL-40 with inflammatory markers were found, supporting the positive association of reactive astrocyte markers with inflammatory proteins in the CSF. A protein-protein interaction network confirmed that GFAP and YKL-40 proteins were inter-connected with the majority (19 out of 21) of the inflammation-related proteins (Supplementary Fig. 7C).

## Discussion

Our results suggest that the two most widely used reactive astrocyte biomarkers, CSF GFAP and YKL-40, are differently associated with AD pathophysiological hallmarks in living humans. While GFAP levels mainly reflect a response to Aβ pathology, YKL-40 levels mainly reflect a response to tau pathology. We demonstrated that these reactive astrocyte biomarkers mediate the effects of Aβ and tau pathologies on hippocampal atrophy and cognitive impairment. Furthermore, we found that CSF GFAP and YKL-40 levels were closely related to the levels of CSF inflammation-related proteins.

In the present study, CSF GFAP levels were associated with Aβ burden in typical AD brain regions, whereas no association was found with tau tangles accumulation. Importantly, we replicated these findings using plasma GFAP, which has been suggested to outperform CSF GFAP in the early detection of Aβ pathology [16]. It has already been reported that astrocytes assume multiple reactive phenotypes, overexpressing specific proteins depending on the pathological stimuli [8, 11]. Previous *post-mortem* observations suggested that reactive astrocytes overexpressing GFAP are found in the vicinity of Aβ plaques [1]. Furthermore, it was reported that the topography of GFAP-immunopositive astrocytes resembles the distribution of Aβ plaques in AD [48]. From the perspective of fluid biomarkers, GFAP levels are elevated in individuals within the AD spectrum [12, 15, 16, 49–51] and highly correlate with Aβ markers [15–17, 49–51]. Altogether, these findings support the notion that astrocytes overexpress GFAP in response to brain Aβ deposition in AD.

Even though YKL-40 is involved in the activation of innate immune cells, its function in the central nervous system remains poorly understood [8, 11]. Importantly, the expression of this protein in the brain tissue has been detected in astrocytes, microglial cells, and infiltrating macrophages [52–54]. However, recent pathological evidence showed that YKL-40 strongly colocalizes with GFAP (astrocyte marker) but not with MAP2 (neuronal marker) and IBA-1 (microglia marker) in the AD brain [55]. Furthermore, another investigation reported that YKL-40 immunoreactivity was mainly observed within astrocytes but not within microglial cells in the frontal cortex of AD patients [56]. Thus, CSF YKL-40 is being increasingly accepted as a reactive astrocyte biomarker in AD [11, 12].

We observed that CSF YKL-40 levels were associated with tau but not Aβ pathology, indicating that YKL-40 levels in the CSF reflect an astrocyte response to tau tangles deposition in AD. In vivo studies suggest that CSF YKL-40 levels are elevated in AD [12, 56] and other tauopathies [18, 57, 58], as well as correlate with CSF tau levels [17–20]. Accordingly, recent *post-mortem* studies reported astrocyte overexpression of YKL-40 in AD and non-AD tauopathies *(e.g*., Pick’s disease, corticobasal degeneration, and progressive supranuclear palsy) [55]. Here, we expanded the aforementioned evidence by demonstrating that CSF YKL-40 levels were associated with tau accumulation - but not Aβ - in brain regions typically affected by AD-related tau pathology. Our models also showed that the association between CSF YKL-40 and tau pathology did not depend on plasma or CSF GFAP levels. Altogether, these results support the notion that astrocytes overexpress YKL-40 in response to tau tangles accumulation in AD.

We showed that reactive astrocyte biomarkers mediate the effect of Aβ and tau on neurodegeneration and cognitive impairment. Previous experimental studies demonstrated that reactive astrocytes actively promote neuronal injury [4, 59–62]. Additionally, neuropathological evidence suggests that reactive astrogliosis is more prominent in brain regions more affected by degeneration in AD and other neurodegenerative conditions (e.g., Parkinson’s disease, Huntington’s disease, and multiple sclerosis) [4]. These findings further support the association of astrocyte reactivity with neurodegeneration and cognitive disfunction found in our study.

We found that CSF GFAP and YKL-40 levels were closely associated with several neuroinflammatory proteins previously associated with AD progression, such as CX3CL1 [63], MMP-10 [64], TRAIL [65], HGF [66], CSF-1 [67], and 4E-BP1 [68]. This finding is in agreement with a growing body of evidence showing that reactive astrocytes are major cellular players in the neuroinflammatory response observed in AD [69, 70] and that AD proteinopathies drive astrocyte signatures related to the activation of inflammatory pathways [9]. Interestingly, CSF YKL-40 was associated with additional AD-related inflammatory markers in comparison to CSF GFAP (e.g., IL-8 [71] and CXCL1 [72]). A possible explanation for this result could be the fact that GFAP has a primary structural function [8, 11], while YKL-40 has been suggested to be directly involved in the inflammatory response by activating the innate immune system [11]. Taken together, these results suggest that the use of in vivo biomarkers of astrocyte reactivity can help to elucidate the complex link between AD hallmark proteins and neuroinflammation.

Our findings have important implications in the context of emerging anti-Aβ and anti-tau therapies for AD [73, 74]. Given that AD is a complex and multifaceted disease [11], it is reasonable to postulate that the combination of therapies - rather than single-target treatments - can offer more effective outcomes. A recent consensus reinforced the importance of developing astrocyte-based therapies for neurodegenerative conditions, suggesting that studies focusing on the characterization of in vivo astrocyte biomarkers should be a priority in order to achieve this goal [8]. In line with this, our results suggest that drugs targeting specific reactive astrocyte phenotypes could potentially be used in combination with anti-Aβ and anti-tau therapies to enhance treatment response in future disease-modifying clinical trials.

This study has methodological limitations. We used two pre-selected biomarkers to test the association between reactive astrogliosis and AD pathophysiology. In this context, other reactive astrocyte biomarkers that were not investigated in the current study could be more closely related to Aβ and tau pathologies than GFAP and YKL-40. Given that we used fluid levels of GFAP and YKL-40, it is also important to acknowledge the lack of topographical information provided by these biomarkers to assess reactive astrogliosis. Some individuals in our study had a time lag of 3–12 months between imaging and CSF collection. Although we cannot exclude that this time interval played a role in our results, results remained unchanged when excluding participants with a time lag higher than 3 months. Our results are phenomenological and do not provide the biological mechanism underlying the associations between AD hallmark proteins and reactive astrocyte biomarkers. However, because it is known that Aβ and tau pathologies induce distinct gene expression signatures in astrocytes [9, 10], our results could be explained by the fact that Aβ leads to a gene expression profile associated with astrocyte overexpression of GFAP, while tau leads to a gene expression profile related to astrocyte overexpression of YKL-40. Future studies are needed to further investigate the biological underpinnings of our results. The use of different approaches to determine the thresholds of biomarker positivity could alter some of our results. For instance, we would have a higher proportion of tau-positive individuals using a meta-ROI confined to the medial temporal cortex [36]. The association of reactive astrocyte biomarkers with brain AD pathophysiology was predominant in the right hemisphere, which should be addressed by subsequent studies with larger sample sizes. Our cohort is composed of individuals that are motivated to participate in a dementia study, potentially being a source of self-selection bias. Lastly, we used a cross-sectional design and modeled AD progressions using individuals across the disease spectrum. It would be highly desirable to replicate our findings using a longitudinal design with multiple time points to better characterize the sequential relation of markers.

To conclude, we observed that plasma and CSF GFAP levels associate with Aβ while CSF YKL-40 levels associate with tau pathology, suggesting the existence of astrocyte biomarker signatures of Aβ and tau tangles in the living AD brain.

## Supplementary information

Supplementary Information

## Data Availability

The data from the TRIAD study will be made available from the senior authors upon reasonable request. Such arrangements are subject to standard data-sharing agreements. Of note, the data used in the present work is not publicly available because the information could compromise the participants’ privacy.

## Code availability

Scripts used for statistical analyses can be provided upon reasonable request.
