# Supplementary material for: Astrocyte biomarker signatures of amyloid-β and tau pathologies in Alzheimer’s disease
Source: Mol Psychiatry. 2022 Aug 10;27(11):4781–9. doi: 10.1038/s41380-022-01716-2 (PMC9734046; doi:10.1038/s41380-022-01716-2)
Supplement: Supplementary file 1 — Supplementary Information [file 41380_2022_1716_MOESM1_ESM.pdf]

Supplementary Information

Article: “Astrocyte biomarker signatures of amyloid-β and tau pathologies in Alzheimer’s disease”

Ferrari-Souza *et al*.

Supplementary Methods. MRI and PET acquisition and processing.

Supplementary Figure 1. Flowchart of included participants.

Supplementary Figure 2. Aβ-PET and tau-PET SUVR average and SD maps.

Supplementary Figure 3. Correlation between reactive astrocyte biomarkers.

Supplementary Figure 4. CSF YKL-40 levels across AT groups, including A-T+ individuals.

Supplementary Figure 5. Voxel-wise linear regression results uncorrected for multiple comparisons correction.

Supplementary Figure 6. Overlap between the regions showing a significant association of CSF GFAP and YKL-40 with Aβ-PET and tau-PET, respectively.

Supplementary Figure 7. GFAP and YKL-40 are associated with CSF inflammatory makers.

Supplementary Table 1. Time differences between imaging modalities.

Supplementary Table 2. Analysis testing whether an interaction between Aβ-PET and tau-PET values was differentially associated with the CDR score in the right and left cerebral hemispheres.

Supplementary Table 3. Analysis testing whether an interaction between Aβ-PET and tau-PET values was differentially associated with memory and executive composite scores in the right and left cerebral hemispheres.

Supplementary Table 4. Associations of CSF GFAP and YKL-40 with neocortical Aβ-PET and temporal meta-ROI tau-PET.

Supplementary Table 5. Sensitivity analyses testing the associations of Aβ-PET and tau-PET with reactive astrocyte biomarkers excluding individuals with a time lag higher than 3 months between imaging and CSF collection.

Supplementary Table 6. Structural equation model coefficients and associated statistics for Figure 3A.

Supplementary Table 7. Structural equation model coefficients and associated statistics for Figure 3B.

Supplementary Table 8. Abbreviations of inflammation-related proteins.

Supplementary Table 9. Demographics of the subset of individuals with available CSF inflammation-related proteins.

Supplementary Table 10. Comparison of demographic information between the whole sample and the subsample with available CSF inflammation-related proteins.

Abbreviations: AD = Alzheimer's disease; ADAD = autosomal dominant Alzheimer's disease; ADNI = Alzheimer's Disease Neuroimaging Initiative; *APOE* ε4 = Apolipoprotein E ε4; Aβ = amyloid-β; CSF = cerebrospinal fluid; CI = cognitively impaired; CU = cognitively unimpaired; GFAP = glial fibrillary acidic protein; MCI = mild cognitive impairment; MMSE = Mini-Mental State Examination; MPRAGE = magnetization prepared rapid acquisition gradient echo; MNI = Montreal Neurological Institute; MRI = magnetic resonance imaging; PET = positron emission tomography; RFT = random field theory; SD = standard deviation; SUVR = standardized uptake value ratio; YKL-40 = chitinase-3-like protein 1.

Supplementary Methods. MRI and PET acquisition and processing.

Structural MRI was acquired at the MNI using a 3-T Siemens Magnetom using a standard head coil. We used the MPRAGE MRI (TR: 2300 ms, TE: 2.96ms) sequence to obtain high-resolution structural images of the whole brain (9° flip angle, coronal orientation perpendicular to the double spin echo sequence, 1x1 mm^2 in-plane resolution of 1 mm slab thickness). Then, the Statistical Parametric Mapping 12 segmentation tool was used to segment anatomical images into probabilistic grey matter and white matter maps. Each grey matter probability map was then non-linearly registered (with modulation) to the ADNI template using DARTEL [1] and voxel values were modulated by multiplying them by the Jacobian determinants derived from the spatial normalization step [2]. MRI images were smoothed using a Gaussian kernel of full width half maximum of 8 mm. Lastly, we visually inspected all images to ensure proper alignment to the ADNI template. Aβ-PET ([^{18}F]AZD4694; 40–70 min post-injection) and tau-PET ([^{18}F]MK-6240; 90–110 min post-injection) scans were acquired on a Siemens High Resolution Research Tomograph. Aβ-PET and tau-PET scans were reconstructed using the ordered subset expectation maximization algorithm on a 4D volume with three frames (3 x 600 seconds) and four frames (4 x 300 seconds), respectively [3]. Then, attenuation correction was performed using a 6-minute transmission scan with a rotating ^{137}Cs point source. Furthermore, PET images were corrected for motion, dead time, decay, and scattered and random coincidences. Following an in-house pipeline, T1-weighted MRI data was corrected for non-uniformity and field distortion. Subsequently, linear co-registration and non-linear spatial normalization for the MNI template was performed through linear and non-linear transformation in two main steps: (i) PET registration to the correspondent T1-weighted MRI, and (ii) T1-weighted MRI registration to the MNI reference space. PET images were spatially smoothed to achieve a final resolution of 8 mm full width at half-maximum width. SUVRs were calculated using the whole cerebellum grey matter reference region for [^{18}F]AZD4694 Aβ-PET [4] and using the inferior cerebellum grey matter as reference region for [^{18}F]MK-6240 tau-PET [5].

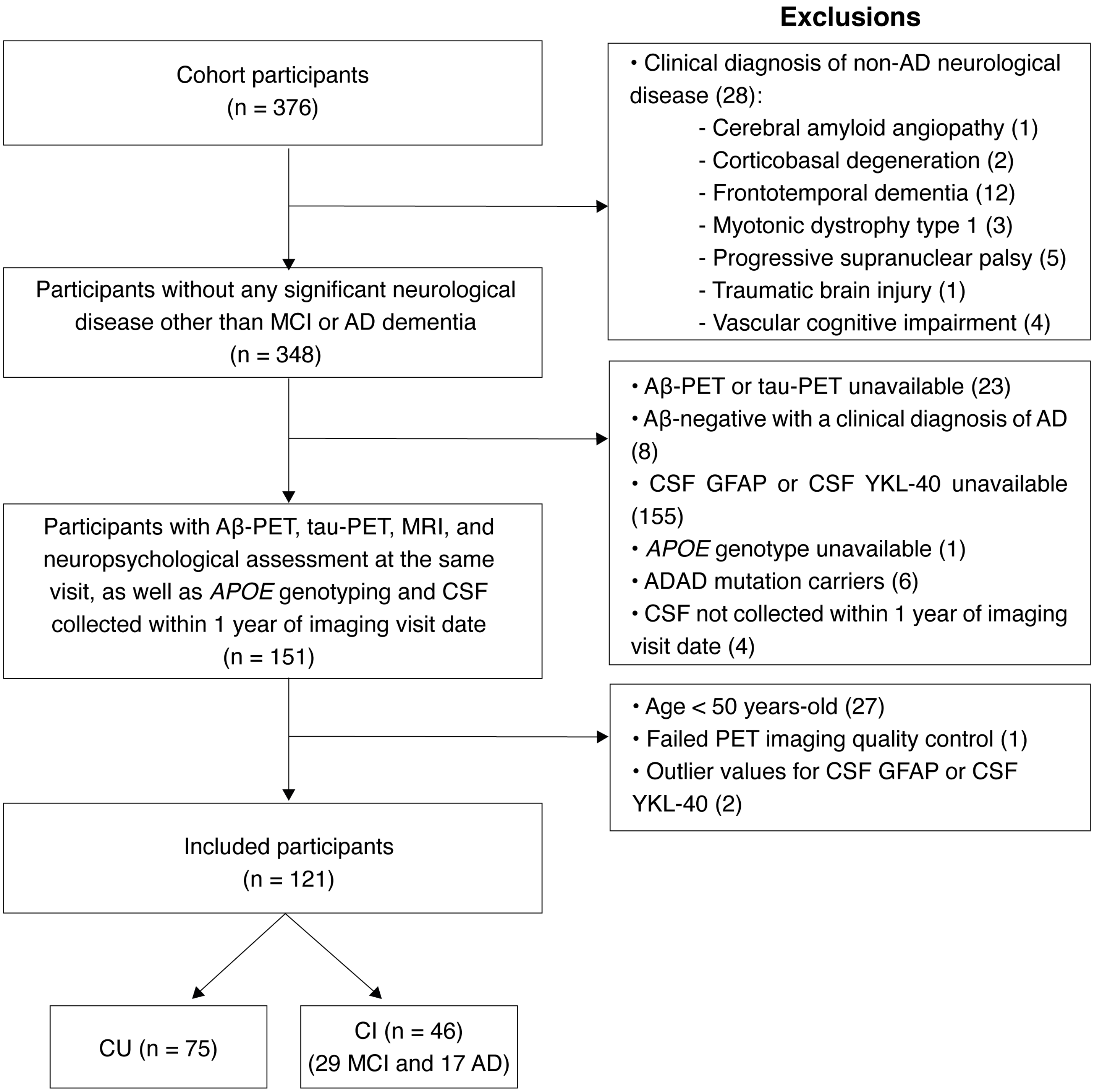

Supplementary Figure 1. Flowchart of included participants.

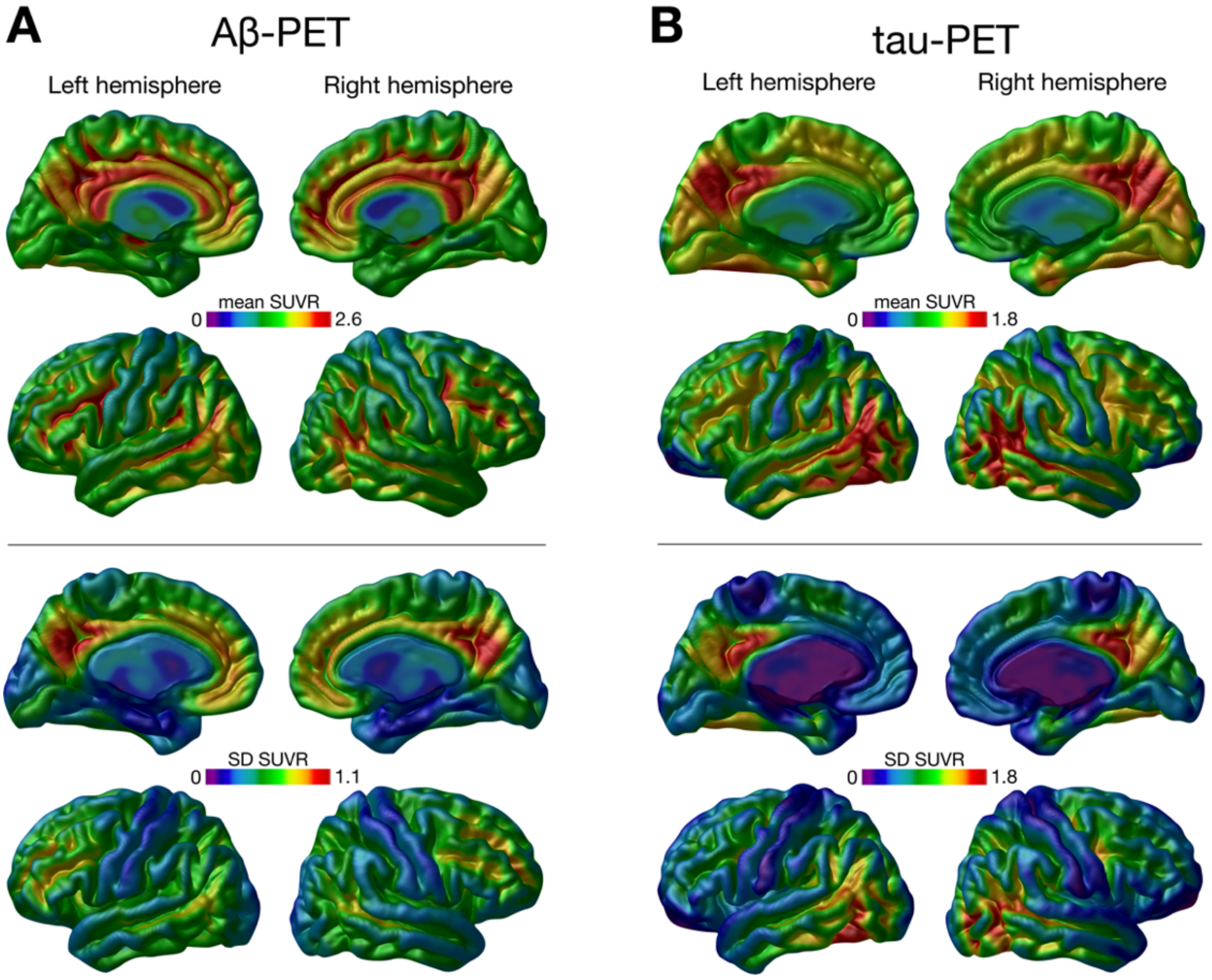

Supplementary Figure 2. Aβ-PET and tau-PET SUVR average and SD maps. Voxel-wise (**A**) Aβ-PET and (**B**) tau-PET SUVR mean (top) and SD (bottom) maps were calculated from the whole study population (75 CU, 29 with MCI, and 17 with AD dementia).

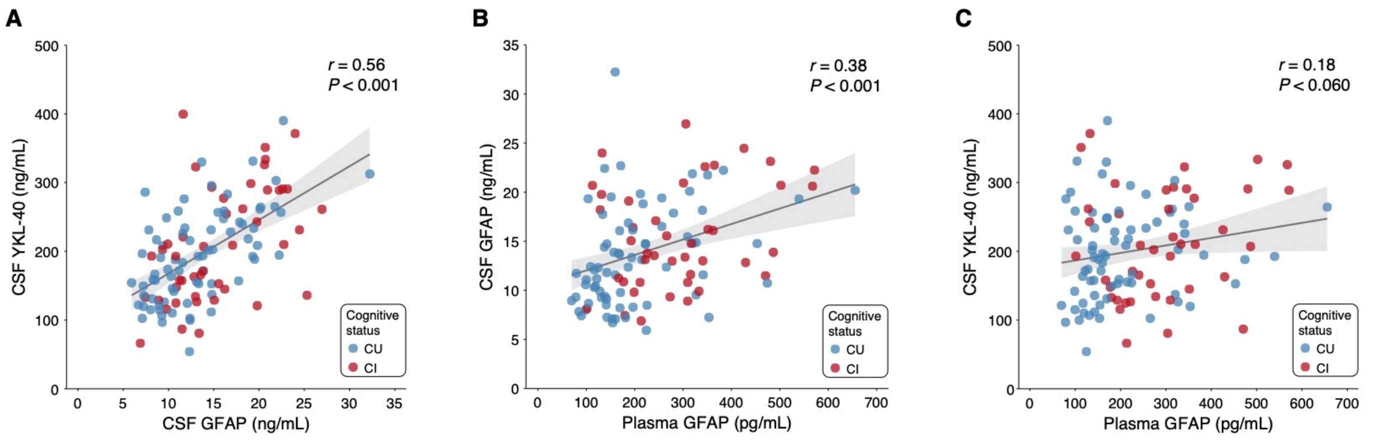

Supplementary Figure 2. Correlation between reactive astrocyte biomarkers. Scatter plot showing the results of Spearman's rank correlations between (**A**) CSF GFAP and CSF YKL-40, (**B**) plasma GFAP and CSF GFAP, and (**C**) plasma GFAP and CSF YKL-40. The error bands indicate the 95% confidence intervals. Individuals are colored by cognitive status. Noteworthy, analyses involving plasma GFAP were conducted in a subset of 114 individuals; from the total study population of 121 subjects, five participants did not have available plasma GFAP measures and two were excluded because they were considered outliers (plasma GFAP concentrations three SD above the mean of the population).

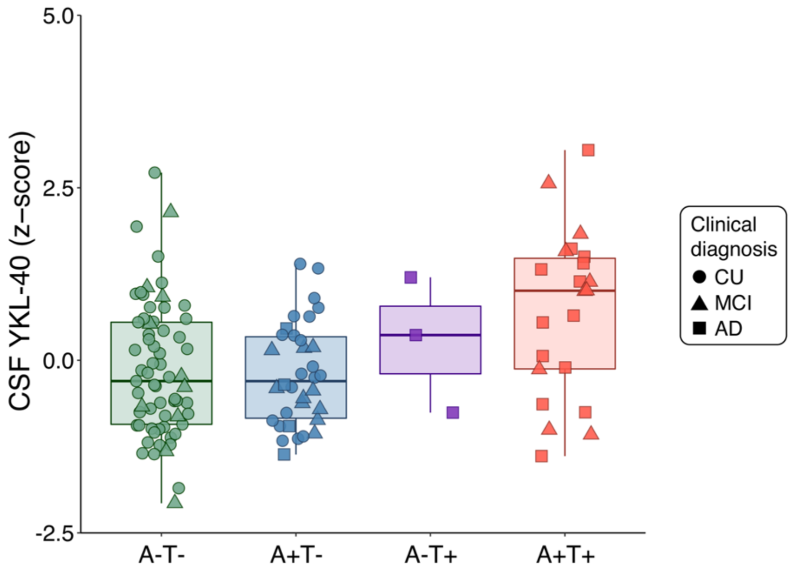

Supplementary Figure 4. CSF YKL-40 levels across AT groups, including A-T+ individuals. Box-and-whisker plot of CSF YKL-40 levels across AT groups. The horizontal line in each box represents the median; box ends represent the 25th and 75th percentiles. CSF YKL-40 levels were adjusted for age and sex; we could not adjust for *APOE* ε4 status as 1 A-T+ individual did not have *APOE* genotype data available. Noteworthy, the 3 A-T+ individuals were not included in our analyses due to exclusion criteria defined *a priori* (Aβ negative subjects with a clinical diagnosis of AD dementia).

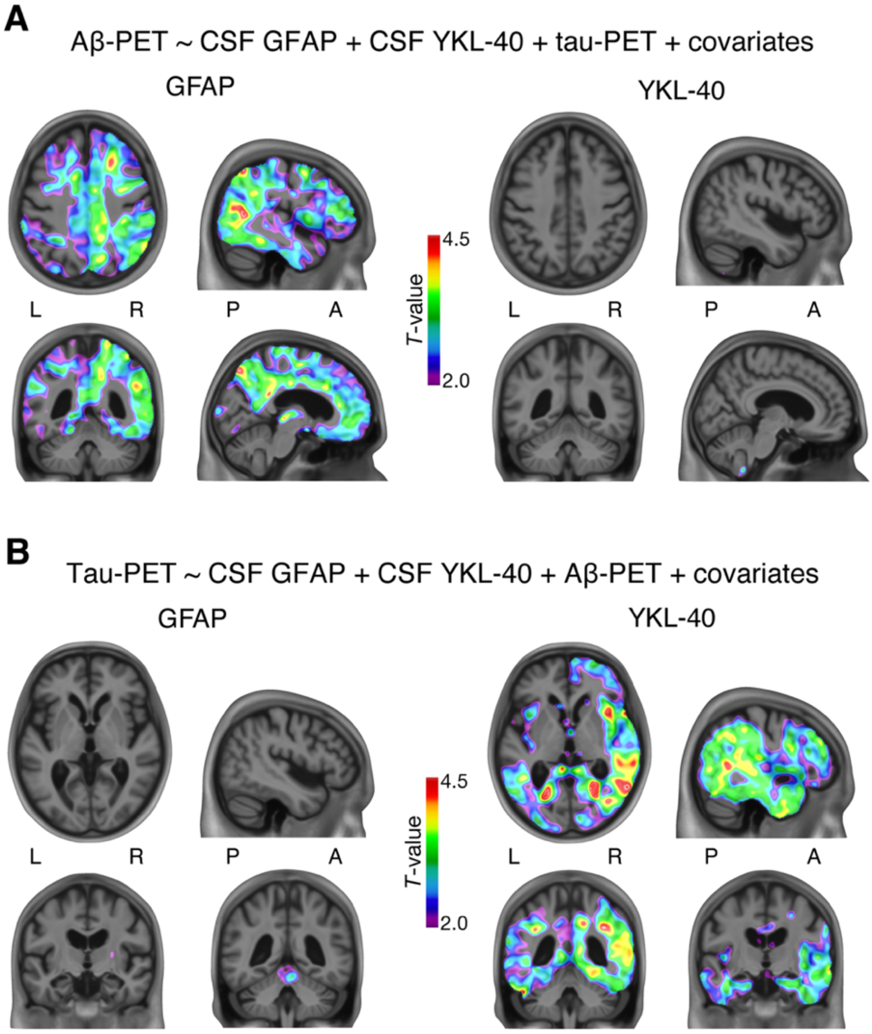

Supplementary Figure 5. Voxel-wise linear regression results uncorrected for multiple comparisons correction. *T*-statistical parametric maps show the result of voxel-wise linear regression testing (**A**) the association of Aβ-PET SUVR with CSF GFAP and YKL-40 levels adjusting for tau-PET SUVR, and (**B**) the association of tau-PET SUVR with CSF GFAP and YKL-40 levels adjusting for neocortical Aβ-PET SUVR. R and L indicate right and left, respectively; A and P denote anterior and posterior, respectively. The results displayed are uncorrected for multiple comparisons correction. Although *T*-statistical parametric maps demonstrated that the relationships of CSF GFAP and YKL-40 with Aβ-PET and tau-PET, respectively, were stronger in the right hemisphere, we observed that the associations were present in both cerebral hemispheres.

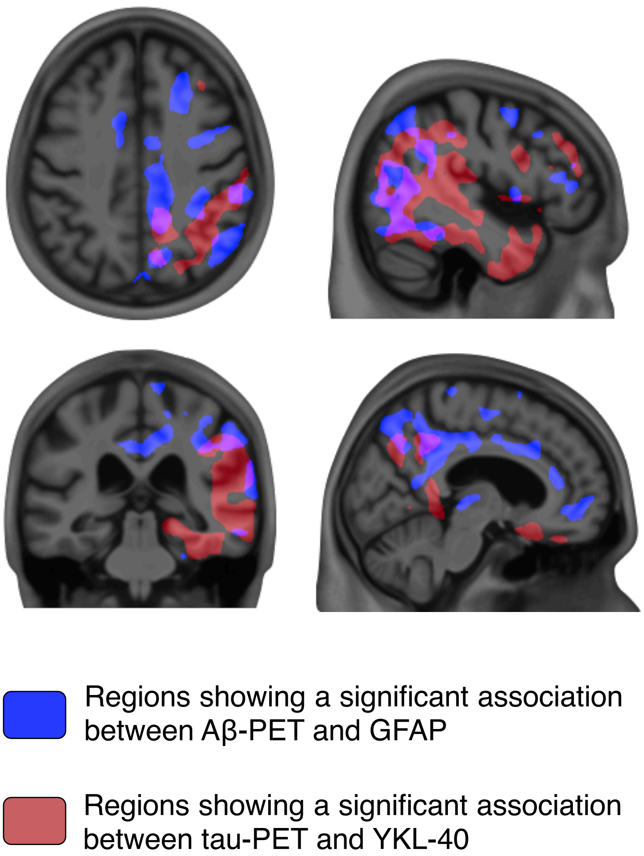

Supplementary Figure 6. Overlap between the regions showing a significant association of CSF GFAP and YKL-40 with Aβ-PET and tau-PET, respectively. The figure depicts the overlap between the regions showing a significant association of CSF GFAP with Aβ-PET (blue) and of YKL-40 with tau-PET (red) from voxel-wise linear regressions. We observed that 78,270 voxels showed a significant association only between CSF GFAP and Aβ-PET, 134,862 voxels showed a significant association only between CSF YKL-40 and tau-PET, and 31,779 voxels showed an overlap between the clusters. The topographical overlap reflects 29% of the area showing an association between CSF GFAP and Aβ-PET and 19% of the area showing an association between CSF YKL- 40 and tau-PET.

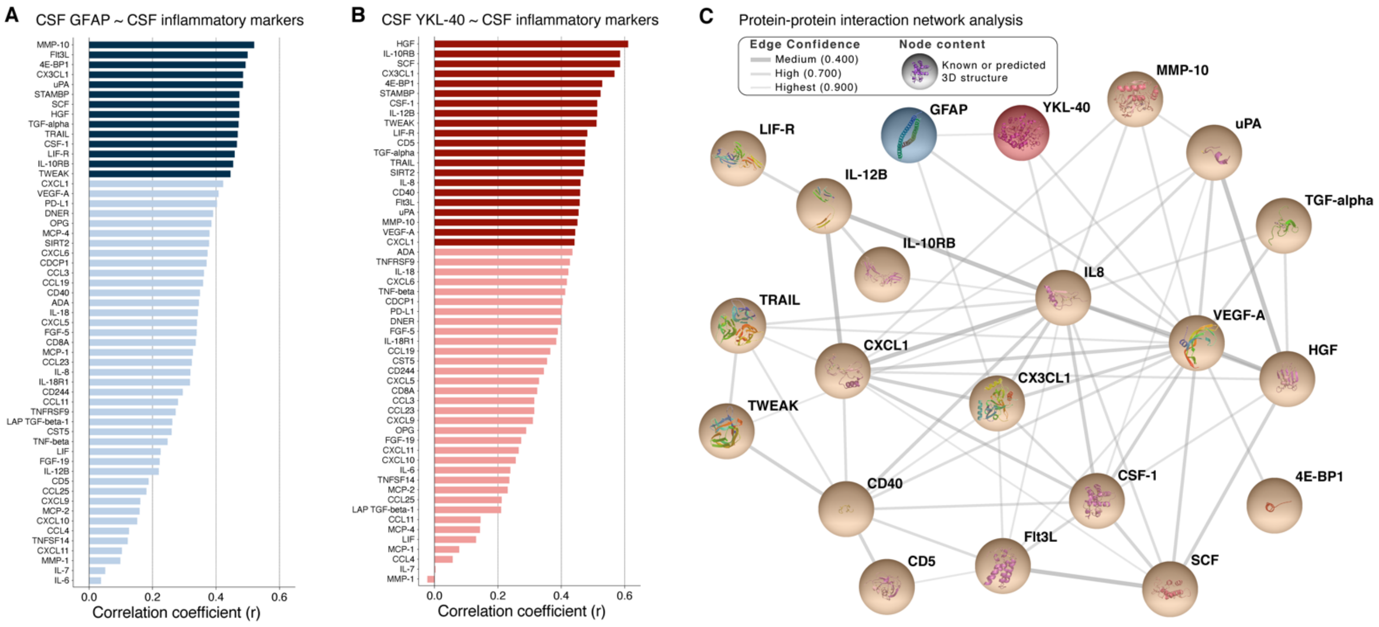

Supplementary Figure 7. GFAP and YKL-40 are associated with CSF inflammatory makers. (A) The graph shows the correlations coefficients from age-adjusted partial correlations between CSF GFAP and CSF inflammation-related proteins. Dark blue bars represent statistically significant correlations after Bonferroni correction for multiple comparisons, whereas light blue bars represent correlations that did not survive Bonferroni correction. **(B)** The graph shows the correlations coefficients from age-adjusted partial correlations between CSF YKL-40 and CSF inflammation-related proteins. Dark red bars represent statistically significant correlations after Bonferroni correction for multiple comparisons, whereas light red bars represent correlations that did not survive Bonferroni correction. (**C**) Protein-protein interaction network analysis further supports that GFAP and YKL-40 were inter-connected with several inflammation-related proteins. STAMBP and SIRT2 were disconnected to the other proteins in the network analysis; thus, these proteins were not represented in the figure. Abbreviations of inflammation-related proteins are reported in **Supplementary Table 6**.

Supplementary Table 1. Time differences between imaging modalities.

	**Mean (SD) time difference, months**
MRI and Aβ-PET	1.04 (1.26)
MRI and tau-PET	1.45 (1.98)
Aβ-PET and tau-PET	1.43 (1.56)

Supplementary Table 2. Analysis testing whether an interaction between Aβ-PET and tau-PET values was differentially associated with the CDR score in the right and left cerebral hemispheres.

	Left hemisphere		Right hemisphere	
	**Odds Ratio (95% CI)**	***P*-value**	**Odds Ratio (95% CI)**	***P*-value**
**CDR ~ neocortical Aβ-PET SUVR x temporal meta-ROI tau-PET SUVR + covariates[a]**				
Neocortical Aβ-PET SUVR	1.69 (0.95 to 3.02)	0.074	1.75 (0.995 to 3.11)	0.054
Temporal meta-ROI tau-PET SUVR	6.20 (2.34 to 19.28)	0.001	7.29 (2.61 to 24.81)	< 0.001
Neocortical Aβ-PET SUVR x temporal meta-ROI tau-PET SUVR	0.65 (0.32 to 1.24)	0.190	0.79 (0.40 to 1.61)	0.491

Ordinal logistic regression demonstrated that the interaction between global neocortical Aβ-PET and temporal meta-ROI tau-PET SUVR was not significantly associated with the CDR score in either cerebral hemisphere ($P \geq 0.190$).

[a]Potential confounders included in the models as covariates are the following: age, sex, and *APOE* ε4 status.

Supplementary Table 3. Analysis testing whether an interaction between Aβ-PET and tau-PET values was differentially associated with memory and executive composite scores in the right and left cerebral hemispheres.

	Left hemisphere		Right hemisphere	
	**β (95% CI)**	***P*-value**	**β (95% CI)**	***P*-value**
**Memory composite ~ neocortical Aβ-PET SUVR x temporal meta-ROI tau-PET SUVR + covariates[a]**				
Neocortical Aβ-PET SUVR	-0.23 (-0.51 to 0.03)	0.082	-0.38 (-0.90 to 0.14)	0.148
Temporal meta-ROI tau-PET SUVR	-0.89 (-1.31 to -0.46)	< 0.001	-1.43 (-2.08 to -0.78)	< 0.001
Neocortical Aβ-PET SUVR x temporal meta-ROI tau-PET SUVR	0.03 (-0.27 to 0.33)	0.847	0.08 (-0.36 to 0.53)	0.717
**Executive composite ~ neocortical Aβ-PET SUVR x temporal meta-ROI tau-PET SUVR + covariates[a]**				
Neocortical Aβ-PET SUVR	0.15 (-0.09 to 0.38)	0.218	0.13 (-0.09 to 0.35)	0.256
Temporal meta-ROI tau-PET SUVR	-0.64 (-1.00 to -0.28)	< 0.001	-0.64 (-0.99 to -0.28)	< 0.001
Neocortical Aβ-PET SUVR x temporal meta-ROI tau-PET SUVR	0.02 (-0.23 to 0.27)	0.881	-0.002 (-0.25 to 0.24)	0.987

Linear regression models revealed that the interaction between global neocortical Aβ-PET and temporal meta-ROI tau-PET SUVR was not significantly associated with memory and executive composite scores in either cerebral hemispheres ($P \geq 0.717$).

[a]Potential confounders included in the models as covariates are the following: age, sex, and *APOE* ε4 status.

Supplementary Table 4. Associations of CSF GFAP and YKL-40 with neocortical Aβ-PET and temporal meta-ROI tau-PET.

	β (95% confidence interval)	*T*-value	*P*-value
**Model A[a]: neocortical Aβ-PET SUVR ~ CSF GFAP + CSF YKL-40 + temporal meta-ROI tau-PET SUVR + covariates[c]**			
CSF GFAP	0.24 (0.08 to 0.40)	3.03	0.003
CSF YKL-40	-0.14 (-0.30 to 0.03)	-1.66	0.100
Temporal meta-ROI tau-PET SUVR	0.53 (0.33 to 0.72)	5.38	< 0.001
**Model B[b]: temporal meta-ROI tau-PET SUVR ~ CSF GFAP + CSF YKL-40 + neocortical Aβ-PET SUVR + covariates[c]**			
CSF GFAP	-0.08 (-0.22 to 0.06)	-1.17	0.244
CSF YKL-40	0.24 (0.11 to 0.37)	3.55	< 0.001
Neocortical Aβ-PET SUVR	0.39 (0.24 to 0.53)	5.38	< 0.001

[a]Adjusted R^2: 0.51. [b]Adjusted R^2: 0.64. [c]Potential confounders included in the models as covariates are the following: age, sex, cognitive status, and *APOE* ε4 status.

Supplementary Table 5. Sensitivity analyses testing the associations of Aβ-PET and tau-PET with reactive astrocyte biomarkers excluding individuals with a time lag higher than 3 months between imaging and CSF collection.

	β (95% confidence interval)	*T*-value	*P*-value
**Model A[a]: neocortical Aβ-PET SUVR ~ CSF GFAP + CSF YKL-40 + temporal meta-ROI tau-PET SUVR + covariates[c]**			
CSF GFAP	0.27 (0.05 to 0.49)	2.46	0.016
CSF YKL-40	-0.17 (-0.40 to 0.07)	-1.37	0.173
Temporal meta-ROI tau-PET SUVR	0.58 (0.32 to 0.84)	4.44	< 0.001
**Model B[b]: temporal meta-ROI tau-PET SUVR ~ CSF GFAP + CSF YKL-40 + neocortical Aβ-PET SUVR + covariates[c]**			
CSF GFAP	-0.17 (-0.35 to 0.01)	-1.90	0.061
CSF YKL-40	0.33 (0.15 to 0.50)	3.69	< 0.001
Neocortical Aβ-PET SUVR	0.36 (0.20 to 0.52)	4.44	< 0.001

[a]Adjusted R^2: 0.48. [b]Adjusted R^2: 0.65. [c]Potential confounders included in the models as covariates are the following: age, sex, cognitive status, and *APOE* ε4 status.

Supplementary Table 6. Structural equation model coefficients and associated statistics for Figure 3A.

	β (95% confidence interval)	*P*-value
**MMSE**		
Hippocampal volume	0.28 (0.12 to 0.43)	0.001
Temporal meta-ROI tau-PET SUVR	-0.48 (-0.68 to -0.28)	< 0.001
Neocortical Aβ-PET SUVR	0.04 (-0.14 to 0.21)	0.690
CSF GFAP	-0.02 (-0.16 to 0.12)	0.762
**Hippocampal volume**		
Temporal meta-ROI tau-PET SUVR	-0.46 (-0.68 to -0.24)	< 0.001
Neocortical Aβ-PET SUVR	-0.03 (-0.23 to 0.17)	0.783
CSF GFAP	-0.16 (-0.32 to -0.003)	0.046
**CSF GFAP**		
Temporal meta-ROI tau-PET SUVR	0.11 (-0.13 to 0.36)	0.361
Neocortical Aβ-PET SUVR	0.30 (0.09 to 0.52)	0.007
**Temporal meta-ROI tau-PET SUVR**		
Neocortical Aβ-PET SUVR	0.58 (0.45 to 0.70)	< 0.001

All associations were adjusted for age, *APOE* ε4 status, and years of education.

Supplementary Table 7. Structural equation model coefficients and associated statistics for Figure 3B.

	β (95% confidence interval)	*P*-value
**MMSE**		
Hippocampal volume	0.30 (0.15 to 0.46)	< 0.001
Temporal meta-ROI tau-PET SUVR	-0.51 (-0.72 to -0.31)	< 0.001
Neocortical Aβ-PET SUVR	0.03 (-0.14 to 0.20)	0.706
CSF YKL-40	0.09 (-0.05 to 0.23)	0.198
**Hippocampal volume**		
Temporal meta-ROI tau-PET SUVR	-0.41 (-0.63 to -0.18)	< 0.001
Neocortical Aβ-PET SUVR	-0.08 (-0.27 to 0.11)	0.418
CSF YKL-40	-0.17 (-0.33 to -0.01)	0.041
**CSF YKL-40**		
Temporal meta-ROI tau-PET SUVR	0.44 (0.20 to 0.67)	< 0.001
Neocortical Aβ-PET SUVR	-0.02 (-0.24 to 0.20)	0.855
**Temporal meta-ROI tau-PET SUVR**		
Neocortical Aβ-PET SUVR	0.58 (0.45 to 0.70)	< 0.001

All associations were adjusted for age, *APOE* ε4 status, and years of education.

Supplementary Table 8. Abbreviations of inflammation-related proteins.

Protein symbol	Protein name
4E-BP1	Eukaryotic translation initiation factor 4E-binding protein 1
ADA	Adenosine Deaminase
CCL11	C-X-C motif chemokine 11
CCL19	C-C motif chemokine 19
CCL23	C-C motif chemokine 23
CCL25	C-C motif chemokine 25
CCL3	C-C motif chemokine 3
CCL4	C-C motif chemokine 4
CD244	Natural killer cell receptor 2B4
CD40	CD40L receptor
CD5	T-cell surface glycoprotein CD5
CD8A	T-cell surface glycoprotein CD8 alpha chain
CDCP1	CUB domain-containing protein 1
CSF-1	Macrophage colony-stimulating factor 1
CST5	Cystatin D
CX3CL1	Fractalkine
CXCL1	C-X-C motif chemokine 1
CXCL10	C-X-C motif chemokine 10
CXCL11	C-X-C motif chemokine 11
CXCL5	C-X-C motif chemokine 5
CXCL6	C-X-C motif chemokine 6
CXCL9	C-X-C motif chemokine 9
DNER	Delta and Notch-like epidermal growth factor-related receptor
FGF-19	Fibroblast growth factor 19
FGF-5	Fibroblast growth factor 5
Flt3L	FMS-related tyrosine kinase 3 ligand
HGF	Hepatocyte growth factor
IL-10RB	Interleukin-10 receptor subunit beta

IL-12B	Interleukin-12 subunit beta
IL-18	Interleukin-18
IL-18R1	Interleukin-18 receptor 1
IL-6	Interleukin-6
IL-7	Interleukin-7
IL-8	Interleukin-8
LAP TGF-beta-1	Latency-associated peptide transforming growth factor beta-1
LIF	Leukemia inhibitory factor
LIF-R	Leukemia inhibitory factor receptor
MCP-1	Monocyte chemotactic protein 1
MCP-2	Monocyte chemotactic protein 2
MCP-4	Monocyte chemotactic protein 4
MMP-1	Matrix metalloproteinase-1
MMP-10	Matrix metalloproteinase-10
OPG	Osteoprotegerin
PD-L1	Programmed cell death 1 ligand 1
SCF	Stem cell factor
SIRT2	SIR2-like protein 2
STAMBP	STAM-binding protein
TGF-alpha	Transforming growth factor alpha
TNF-beta	TNF-beta
TNFRSF9	Tumor necrosis factor receptor superfamily member 9
TNFSF14	Tumor necrosis factor ligand superfamily member 14
TRAIL	TNF-related apoptosis-inducing ligand
TWEAK	Tumor necrosis factor ligand superfamily, member 12
uPA	Urokinase-type plasminogen activator
VEGF-A	Vascular endothelial growth factor A

Supplementary Table 9. Demographics of the subset of individuals with available CSF inflammation-related proteins.

	CU	CI	*P*-value
No.	36	26	-
Age, years	71.6 (6.1)	69.6 (7.9)	0.296
Male, No. (%)	9 (25.0)	16 (61.5)	0.008
Education, years	14.5 (3.5)	15.6 (2.8)	0.174
APOE ε4 carriers, No. (%)	11 (30.6)	16 (61.5)	0.030
MMSE score	29.2 (1.1)	26.6 (3.9)	0.002
Neocortical Aβ-PET SUVR	1.62 (0.5)	2.30 (0.5)	< 0.001
Temporal meta-ROI tau-PET SUVR	0.87 (0.1)	1.54 (0.7)	< 0.001
Hippocampal volume, cm^3	3.53 (0.3)[a]	3.24 (0.4)[a]	0.004

Continuous variables are presented as mean (SD).

[a]Values reported are adjusted for total intracranial volume.

Supplementary Table 10. Comparison of demographic information between the whole sample and the subsample with available CSF inflammation-related proteins.

	Whole sample	Subsample with available CSF inflammatory markers	*P*-value
No.	121	62	-
Age, years	70.2 (6.6)	70.8 (6.9)	0.569
Male, No. (%)	53 (43.8)	25 (40.3)	0.770
Education, years	14.9 (3.3)	15.0 (3.2)	0.934
Clinical diagnosis, No. (%)			
CU	75 (62.0)	36 (58.1)	
MCI	29 (24.0)	17 (27.4)	0.859
AD	17 (14.0)	9 (14.5)	
APOE ε4 carriers, No. (%)	48 (39.7)	27 (43.5)	0.729
MMSE score	27.6 (3.9)	28.1 (2.9)	0.352
Neocortical Aβ-PET SUVR	1.77 (0.6)	1.90 (0.6)	0.167
Temporal meta-ROI tau-PET SUVR	1.14 (0.6)	1.15 (0.6)	0.874
Hippocampal volume, cm^3	3.39 (0.4)[a]	3.41 (0.4)[a]	0.787

Continuous variables are presented as mean (SD).

[a]Values reported are adjusted for total intracranial volume.

Supplementary References

1. Ashburner J. A fast diffeomorphic image registration algorithm. Neuroimage. 2007;38(1):95-113.

2. Ashburner J, Friston KJ. Voxel-based morphometry--the methods. Neuroimage. 2000;11(6 Pt 1):805-21.

3. Pascoal TA, Shin M, Kang MS, Chamoun M, Chartrand D, Mathotaarachchi S, et al. In vivo quantification of neurofibrillary tangles with [(18)F]MK-6240. Alzheimers Res Ther. 2018;10(1):74.

4. Cselenyi Z, Jonhagen ME, Forsberg A, Halldin C, Julin P, Schou M, et al. Clinical validation of 18F-AZD4694, an amyloid-beta-specific PET radioligand. J Nucl Med. 2012;53(3):415-24.

5. Pascoal TA, Therriault J, Benedet AL, Savard M, Lussier FZ, Chamoun M, et al. 18F-MK-6240 PET for early and late detection of neurofibrillary tangles. Brain. 2020;143(9):2818-30.
